# Spatial transcriptional mapping reveals the molecular characteristics of juxtaglomerular cell tumors

**DOI:** 10.3389/fonc.2026.1768296

**Published:** 2026-04-10

**Authors:** Jia Li, Hui Luo, Yangcai Wang, Jingzhen Zhu, Jiang Zhao, Bishao Sun, Zhenxing Yang, Ji Zheng, Yajun Song, Zhenqiang Fang

**Affiliations:** 1 Department of Urology, Second Affiliated Hospital, Army Medical University, Chongqing, China; 2 Health Management Department, Second Affiliated Hospital, Army Medical University, Chongqing, China; 3 Hematopoietic Acute Radiation Syndrome Medical and Pharmaceutical Basic Research Innovation Center, Ministry of Education of the People’s Republic of China, Chongqing, China

**Keywords:** gene expression, juxtaglomerular cell tumor, *KISS1*, molecular characteristic, spatial transcription sequencing

## Abstract

**Background:**

Aside from renin expression, little is known about the molecular markers or signaling pathways involved in the pathogenesis of Juxtaglomerular Cell Tumors (JGCTs). This study aimed to elucidate the molecular characteristics underlying JGCTs.

**Methods:**

An 8-year-old girl diagnosed with JGCT underwent laparoscopic partial nephrectomy. Paraffin-embedded tumor tissues were obtained for Spatial Transcriptomic sequencing (STs). The publicly available TCGA database was used to compare the gene expression profiles with those from STs.

**Results:**

The patient presented with typical clinical manifestations of JGCTs. Spatial transcriptomic analysis revealed that juxtaglomerular cells were surrounded by dendritic, endothelial, mast cells, and luminal cells. High expression of *REN*, *KISS1*, *NOTCH3*, *CD34*, *VIM*, and *GATA3* was detected in JGCT tissues. *REN* expression was positively correlated with *KISS1* expression both in JGCTs and other tumor types, including THYM, THCA, TGCT, and KIRP. In tumor samples, high *REN* expression was associated with poorer survival outcomes in THYM, KIRP, BRCA-LumA, and ACC. Co-expression network analysis indicated that *REN*, *KISS1*, and *PPARG* regulate endocrine signaling pathways.

**Conclusion:**

Spatial transcriptomics revealed that high KISS1 expression plays a key role in JGCT development. In addition, the PPARG, NOTCH, and PDGFB pathways were identified as potential regulators of renin secretion.

## Introduction

Juxtaglomerular cell tumor (JGCT), also known as reninoma, is a rare kidney neoplasm characterized by renin secretion, elevated aldosterone levels, hypertension, and hypokalemia. The tumor was first described in 1967 (1). It occurs most frequently in young adults but can be diagnosed later in life. Although no large-scale epidemiological studies have investigated JGCT prevalence, fewer than 261 cases have been reported in a systematic review (2). No data are available regarding the incidence of JGCTs in China. The tumor is generally considered benign; however, five cases have demonstrated distant metastasis and local recurrence involving organs, such as the kidney, liver, lung, and thoracic great vessels (3).The most severe clinical manifestation is secondary persistent hypertension accompanied by hypokalemia, caused by renin secretion. This condition can be corrected by surgical tumor resection (4). Surgically, JGCTs have been treated with open partial nephrectomy and laparoscopic partial nephrectomy. Partial nephrectomy preserves ipsilateral renal function, and its use is gradually increasing. With the advancement and application of laparoscopic techniques, laparoscopic partial nephrectomy, a minimally invasive approach, is becoming the mainstream surgical option (5).

Immunohistological staining indicates that the juxtaglomerular cells express renin, CD34, smooth muscle actin, CD138, vimentin, and collagen IV, while they are negative for cytokeratins, S100, c-Kit, and desmin (6). However, these markers are commonly positive in both JGCTs and glomerulomas, which may exhibit similar morphological features; thus, their diagnostic specificity remains controversial. In addition, CD117 positivity may indicate infiltrating trypsin-positive mast cells (7). Recent studies have suggested that GATA3 could serve as an alternative diagnostic marker for JGCTs, although molecular biological evidence is lacking. These questions remain to be addressed. To identify the genes, signaling pathways, and molecular features that characterize human JGTCs, we performed spatial transcriptomic sequencing (STs).

## Methods

### Samples

An 8-year-old girl diagnosed with a juxtaglomerular cell tumor underwent laparoscopic partial nephrectomy at the Department of Urology, Army Medical University, Chongqing, China. Tumor tissue was obtained and paraffin-embedded for subsequent analysis. Detailed diagnostic and treatment information is presented in the Results section. This study was approved by the Ethics Committee of the Army Medical University in accordance with the Declaration of Helsinki. Written informed consent was obtained from the patient’s guardian.

### Spatial transcriptomic sequencing procedure

Formalin-fixed, paraffin-embedded (FFPE) tissues were stored at 4 °C to preserve RNA integrity. RNA quality was assessed using the Agilent 2100 Bioanalyzer with the 2100 RNA 6000 Nano kit. The RNA concentration was 456.84 ng/μl, with an OD 260/280 ratio of 2.04 and a DV200 value of 56% prior to sectioning onto Visium HD Spatial slides.

Tissue sections (5 μm) were placed on Superfrost™ Plus Microscope Slides (Fisherbrand™), deparaffinized, and stained with H&E following the Visium HD FFPE Tissue Preparation Handbook (CG000684). After decrosslinking, slides were processed on a Visium CytAssist instrument to transfer analytes to a Visium HD Spatial Gene Expression slide with a capture area of 0.42 cm².

Transcriptome probe panels were hybridized to pretreated tissues. Following hybridization, ligase was added to seal probe pairs hybridized to RNA, forming ligation products. Single-stranded ligation products were released upon RNase treatment and tissue permeabilization, then captured on Visium HD slides. Spatial barcodes were added, and libraries were prepared. Spatially barcoded ligation products were released, harvested for qPCR, and indexed via sample index PCR. Library molecules were purified with SPRIselect, quantified, and assessed on a Bioanalyzer prior to sequencing on the Illumina NovaSeq platform according to 10x guidelines.

### Data analysis and statistics

Raw sequencing data were processed using the 10x Genomics Space Ranger v3.1 mkfastq pipeline to generate FASTQ files. The software can be obtained at Official 10x Genomics Support (https://www.10xgenomics.com/support/software/space-ranger/latest). Quality Control of the FASTQ file from sequencing was performed by removing low-quality reads and the adapter sequences; adapters were required to match at least 8bp.

Then sequences were mapped to GRCm39 using the Visium Human Probe Set v2.1 with default parameter settings, and gene expression counts were obtained using Space Ranger count. Gene expression analysis was performed using R packages, namely, Seurat (Version 5.3.1) (8). Normalization, dimensionality reduction, clustering, and differential expression analyses were performed. Gene expression levels were quantified as transcripts per million (TPM). Two key parameters (number of RNA features ≥200 and total count of reads >400) were used for filtering the raw cells. The parameter of resolution in cluster finding was determined to be 1. For clustering, highly variable genes were selected, and the principal components based on those genes were used to build a graph. Seurat also provides UMAP and t-SNE dimensional reduction methods for data processing.

The R platform was used for bioinformatic analysis (https://www.r-project.org/). The R package TCGAplot was used to examine the commonalities and heterogeneity among genomic and cellular alterations in diverse types of tumors (9). TIMER3.0 was employed to comprehensively explore tumor immunological, clinical, and genomic features (10). The iTALK software was employed to display ligand-receptor interactions between different cell types (11), providing a computational approach to characterize, compare, and illustrate intercellular communication signals in the multicellular ecosystem. The scMetabolism R package was employed to quantify and visualize metabolic activity at the single-cell level (12).

## Results

### Case presentation and treatment

An 8-year-old girl was admitted to the urology department with paroxysmal syncope, chest tightness, and dyspnea. Physical examination revealed severe hypertension (180/150 mmHg). Laboratory findings included hypokalemia (K^+^: 2.72 mmol/L), a markedly elevated renin level (> 4 ng/mL/h), hyperaldosteronism (plasma aldosterone: 721 pg/mL), and abnormal angiotensin levels (Angiotensin I > 24 ng/mL/h and Angiotensin II: 85.2 pg/mL).

**Figure 1:**
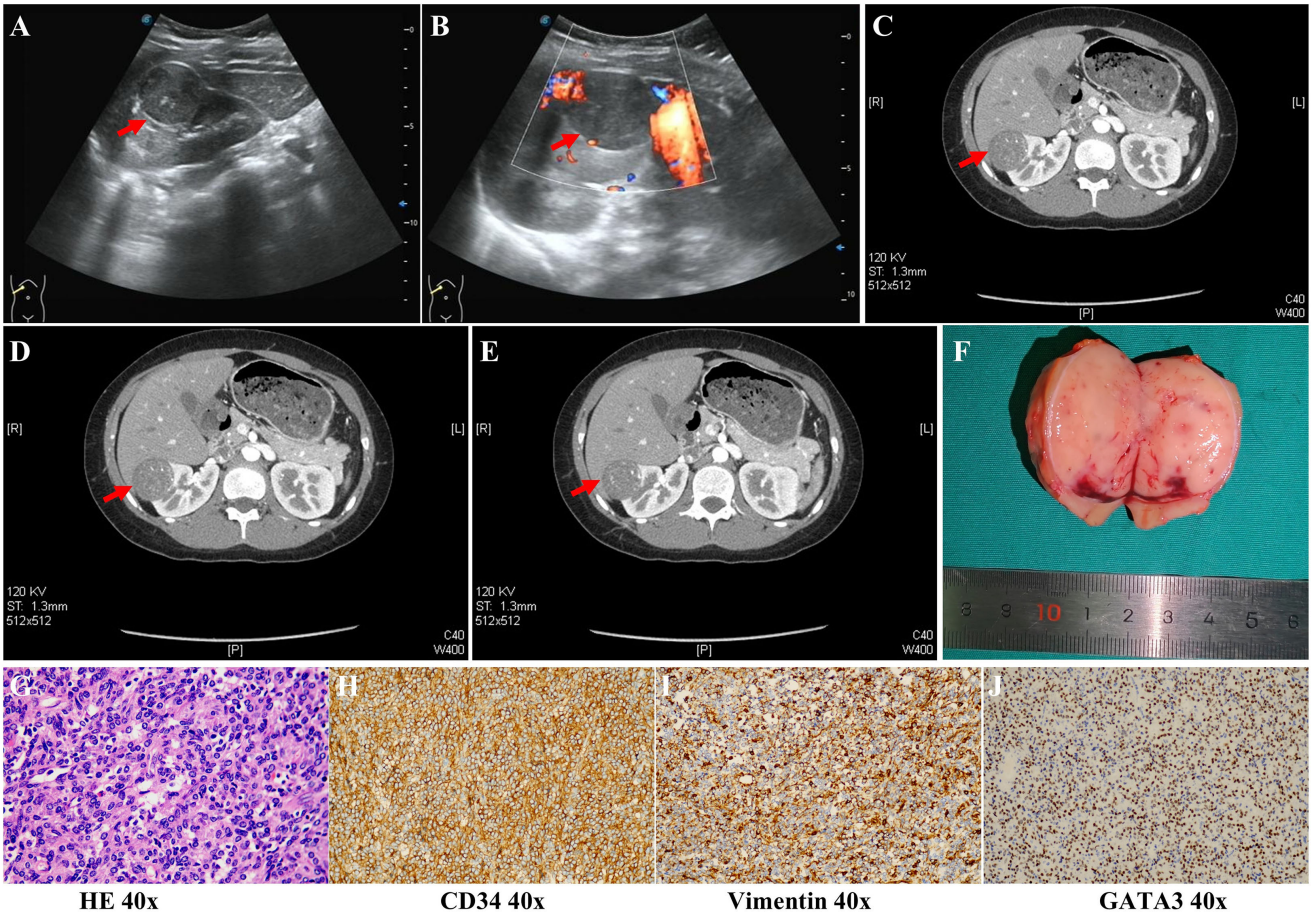
Clinical examination of the patient with JGCT. **(A)** Ultrasound examination shows a hypoechoic spherical mass in the right renal. **(B)** Color Doppler flow imaging (CDFI) shows punctate blood flow signals in the right kidney. **(C-E)** Enhanced CT scans show a single, round or punctate low-density lesion in the renal cortex with slight hemorrhage. **(F)** A solid tumor was obtained from the patient. **(G)** HE staining indicates JGCT tissue. **(H-J)** Immunohistochemical staining of CD34, vimentin, and GATA3 in tumor tissues. Red arrow indicates the location of the tumor in the kidney.

Abdominal ultrasound revealed a normal-sized kidney with clear structure. A hypoechoic spherical mass measuring approximately 3.7 x 3.1 cm was visible beneath the capsule of the right kidney (**Figure 1A**). The mass had a regular shape and clear borders, with a weak echogenic halo. Color Doppler Flow Imaging (CDFI) showed punctate blood flow signals (**Figure 1B**). Abdominal CT demonstrated a vascular tumor in the right renal cortex, appearing as a round or punctate low-density lesion with slight hemorrhage (**Figures 1C–E**). Given the classical clinical features and the ineffective response to multiple oral antihypertensive medications, a diagnosis of JGCT was made.

Further history revealed that the patient had experienced paroxysmal dizziness, chest tightness, and shortness of breath since August 30, 2025, and was initially admitted to Guizhou Provincial People’s Hospital with a blood pressure of 180/150 mmHg. Treatment with phenylbenzamine hydrochloride (1 tablet, twice daily) and amlodipine besylate (half a tablet once daily) was ineffective, and paroxysmal syncope persisted. Five days before admission to our department, she developed frequent nocturia, approximately 4–5 times per night.

Following diagnosis, the patient underwent laparoscopic partial nephrectomy based on tumor size and location. The solid tumor measured approximately 5 cm in diameter with well-defined borders (**Figure 1F**). Postoperatively, blood pressure (101/60 mmHg), serum potassium (3.63 mmol/L), aldosterone (30.1 pg/mL), and renin levels (> 4 ng/mL/h) returned to normal. Pathological examination confirmed the diagnosis of JGCT (**Figures 1G–J**). Immunohistochemistry results were as follows: LCA (–), Ki-67 (5% +), WT-1 (-), PAX-8 (-), CK (-), Vimentin (+), HMB45 (-), S-100 (-), Desmin (-), SMA (-), CD34 (+), GATA3 (+), Inhibin A (-), CD56 (-), H-caldesmon (-), IV-collagen (partially +), NSE (-), Melan A (-), STAT-6 (-), and CD31 (vascular +).

### Molecular characteristic of JGCTs

**Figure 2:**
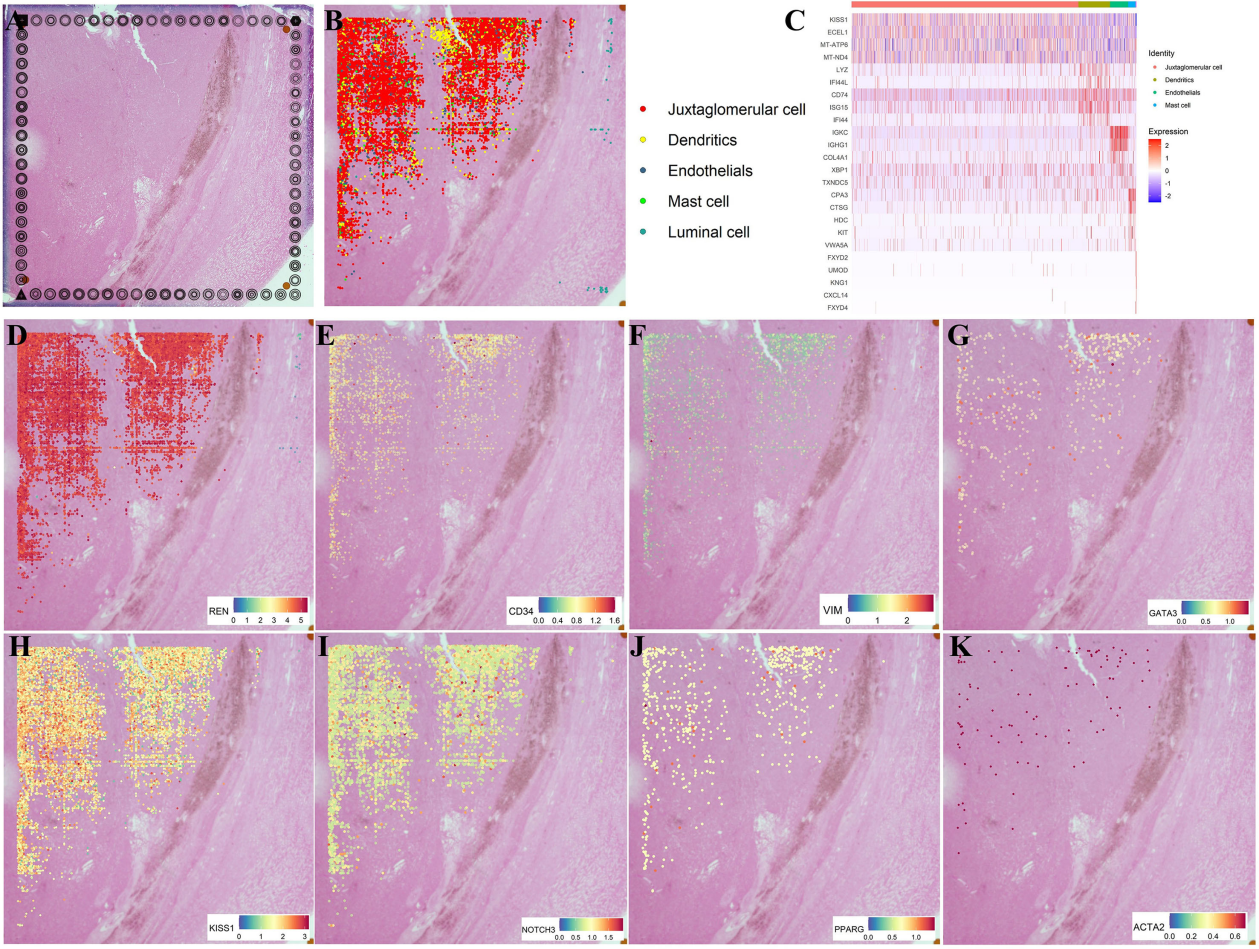
Molecular characteristics of JGCTs revealed by spatial transcriptomic sequencing. **(A)** HE staining image shows the selected region for Spatial Transcriptomic sequencing. **(B)** Spatial distribution and cell types in JGCT tissues. **(C)** Heatmap showing DEGs for each cell type. **(D-K)** Expression of REN, CD34, VIM, GATA3, KISS1, NOTCH3, PPARG, and ACTA2 examined by Spatial Transcriptomics.

To investigate the heterogeneity of juxtaglomerular cells, the immune microenvironment, and their spatial landscape in JGCTs, we collected tumor tissue resections for spatial transcriptomics using the 10x Genomics Visium platform (**Figure 2A**). Five cell types were identified based on high expression of canonical markers:juxtaglomerular cells, dendritic cells, endothelial cells, mast cells, and luminal cells (**Figure 2B**). Consistent with published reports, REN (++++), CD34 (++), VIM (++), and GATA3 (+) were found to be expressed in JGCT tissues (**Figures 2D–G**). In addition, KISS1, NOTCH3, and PPARG were co-expressed in tumor tissues (**Figures 2H–J**). Low expression of α-SMA (encode gene *ACTA2*, **Figure 2K**) was detected, consistent with immunohistochemistry results.

**Figure 3:**
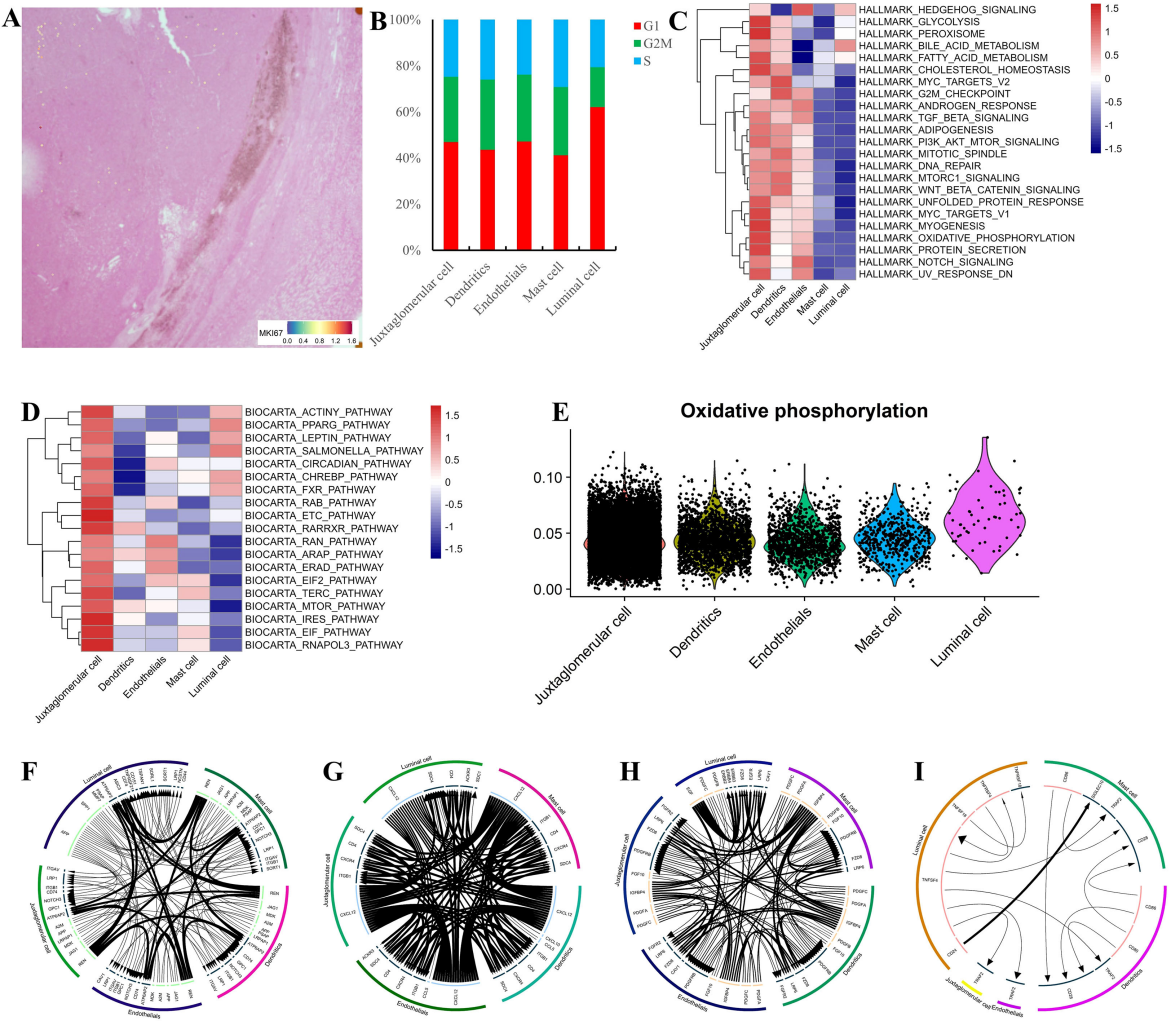
Signaling and cell-cell interactions in JGCTs. **(A)** Expression of MKI67 examined by Spatial Transcriptomic sequencing. **(B)** Cell cycle scores analysis across different JGCT cell types. **(C)** Enrichment analysis of HALLMARK gene sets. Each row represents a gene set, and each column represents a different cell type in JGCTs. **(D)** Enrichment analysis of BIOCARTA gene sets. **(D)** Oxidative phosphorylation signature scores across different JGCT cell types. **(F-I)** Circos plots for a single cohort showing the top 20 highly expressed ligand-receptor interactions in JGCTs by using iTALK.

Given the Ki-67 (5% +) expression, which was confirmed by STs (**Figure 3A**), cell cycle status was assessed by averaging the expression of gene sets representing G, G2M, and S phases. Approximately half of the juxtaglomerular cells were in a proliferative state (**Figure 3B**). Pathway enrichment analysis revealed activation of oxidative phosphorylation, NOTCH signaling, and the PPARG pathway in juxtaglomerular cells (**Figures 3C, D**). GSVA scores for oxidative phosphorylation were evenly distributed across cell types (**Figure 3E**). Highly expressed REN interacted with other cell types via ATPT6AP2 and ASIC3 receptors (**Figure 3F**). CXCL12 signaling occurred primarily through CXCR4 (**Figure 3G**). PDGFB, mainly expressed by endothelial cells, interacted with PDGFRB (**Figure 3H**). No significant checkpoint ligand-receptor interactions were observed (**Figure 3I**). In summary, juxtaglomerular cells highly expressed *REN* and *KISS1*, and their function may be regulated through oxidative phosphorylation, NOTCH signaling, and PPARG pathways. However, the underlying mechanisms require further investigation.

### Analysis of REN expression from the TCGA database

**Figure 4:**
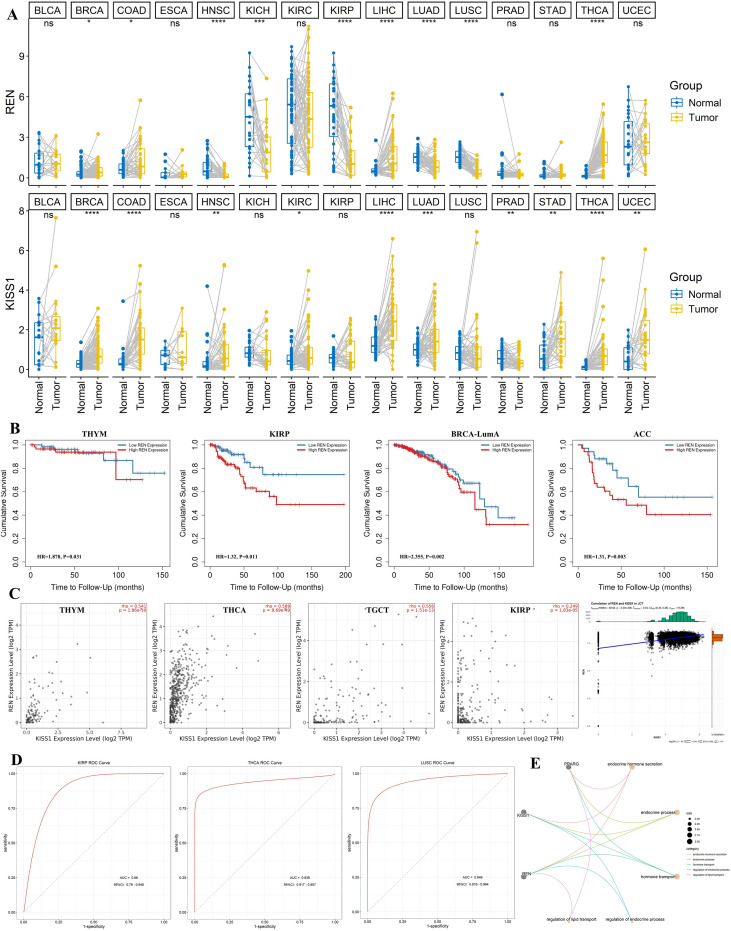
Expression of REN in tumors from the TCGA database. **(A)** Expression of REN and KISS1 in pan-cancer data from TCGA. Yellow denotes tumor samples, and blue represents para-healthy samples. ***p < 0.001, **p < 0.01, *p < 0.05. **(B)** Survival analysis of REN expression in THYM, KIRP, BRCA-LumA, and ACC. **(C)** Pearson correlation of REN and KISS1 expression level (log2TPM) in THYM, THCA, TGCT, and KIRP patients, and also in STs. **(D)** ROC curve analysis showing the diagnostic performance of REN expression in KIRP, THCA, and LUSC patients. ROC, receiver operating characteristic. **(E)** Enrichment network analysis of REN, KISS1, and PPARG genes.

Given the high expression of *REN* and *KISS1* in JGCTs, we analyzed their expression in public TCGA datasets. *REN* and *KISS1* were upregulated in THCA, BRCA, COAD, and LIHC compared to normal tissues (**Figure 4A**). High *REN* expression was associated with poorer survival in THYM (HR = 1.878, P = 0.031), KIRP (HR = 1.32, P = 0.011), BRCA-LumA (HR = 2.355, P = 0.002), and ACC (HR = 1.31, P = 0.003) (**Figure 4B**). In addition, *REN* expression positively correlated with *KISS1* in THYM (rho=0.541, P < 0.001), THCA (rho=0.589, P < 0.001), TGCT (rho=0.5556, P < 0.001), and KIRP (rho=0.249, P < 0.001), and also in STs (R = 0.34, P < 0.001) (**Figure 4C**). ROC curve analysis showed that *REN* expression discriminated tumor from normal tissue with an AUC of 0.88 (95%CI: 0.79-0.948) in KIRP, 0.938 (95%CI: 0.917-0.957) in THCA, and 0.946 (95%CI: 0.919-0.964) in LUSC (**Figure 4D**). Co-expression network analysis indicated that *REN*, *KISS1*, and *PPARG* regulated endocrine signaling pathways (**Figure 4E**).

## Discussion

JGCTs are extremely rare kidney tumors of the juxtaglomerular cells, with fewer than 261 cases reported in the literature. The male-to-female ratio is approximately 2.1:1, with an age range of 6 to 76 years. Patients younger than 20 years, those in the 20–40 years range, and those over 40 years old accounted for 28.86%, 52.96%, and 18.18% of cases, respectively (2). However, patients under 10 years of age are exceedingly rare, with only six cases (three males and three females) reported to date. The present case of an 8-year-old girl is rarely reported in China. The patient exhibited classic clinical features of JGCT, including severe hypertension due to elevated renin levels, hypokalemia, and secondary aldosteronism.

Wilms tumors, which secrete renin in approximately 60% of cases and commonly occur in children, must be considered in the differential diagnosis (13). Wilms tumors exhibit multilayered differentiation with undifferentiated germinal tissue, mesenchymal stroma, and epithelial-like components, often with significant atypia, frequent mitoses, and high WT1 expression but low or absent CD34 or renin (REN) expression (14). In contrast, JGCTs are characterized by cells with round nuclei arranged in nests and abundant eosinophilic cytoplasm, along with diffuse renin positivity and consistent CD34 and vimentin expression, as confirmed by both published studies and our sequencing data (15). Although GATA3 has been proposed as a marker to distinguish JGCTs from other renal tumors, our STs data showed low GATA3 expression (16). Some case reports have described positivity for CD117 (c-kit), Ki-67, and α-smooth muscle actin in JGCTs (17). However, our STs revealed low expression of these markers. Notably, mast cells diffusely distributed throughout JGCT tissues expressed c-kit, suggesting that c-kit positivity observed in immunohistochemistry may originate from infiltrating mast cells rather than tumor cells. Therefore, c-kit should not be used as a diagnostic marker for JGCTs.

The origin of juxtaglomerular cells remains unclear due to rapid differentiation upon removal from the kidney. Some researchers propose derivation from perivascular mesenchymal cells, while others suggest an origin from the juxtaglomerular apparatus. A more widely accepted view is that renin precursor cells can differentiate into juxtaglomerular cells and arteriolar smooth muscle cells, and that juxtaglomerular cells may transdifferentiate into smooth muscle cells (18). Conversely, under conditions requiring increased renin for homeostasis, smooth muscle cells may revert to renin precursor cells. The similarity between arteriolar smooth muscle cells and renin cells may explain the limited smooth muscle differentiation observed in JGCTs, although evidence remains incomplete.

Apart from luminal cells derived from normal adjacent tissue, three immune-related cell types—endothelial cells, dendritic cells, and mast cells—were diffusely distributed within JGCTs. To our knowledge, this is the first description of the immune microenvironment in JGCTs. Cell–cell interaction analysis revealed that CXCL12 (from dendritic cells and mast cells) and PDGFB (platelet-derived growth factor B) may regulate juxtaglomerular cell activation. PDGFB has been reported to reduce renin levels both in the culture medium and intracellularly in renin-synthesizing As4.1 cells (18), but the detailed regulatory mechanisms require further investigation.

Deep RNA sequencing of JGCTs from different patients previously identified 54 co-upregulated genes (19); 17 of these were also highly expressed in our case, namely *REN*, *KISS1*, *SFRP4*, *PTP4A3*, *CSRP2*, *CPE*, *NOTCH3*, *MFGE8*, *JAG1*, *SERPINI1*, *TBX2*, *TPM2*, *SLIT3*, *MXRA8*, *NT5DC2*, *ADCY3*, and *PLEKHH3*. Among these, *KISS1* emerged as a key molecule that was highly expressed in JGCTs and positively correlated with *REN* expression. Previous studies have shown that *KISS1* is the second most abundant transcript in JGCTs and is located adjacent to the *REN* gene on chromosome 1 in both mice and humans (20). We also observed a positive correlation between *REN* and *KISS1* in THYM, THCA, and TGCT, particularly in endocrine-related tumor (21). However, high *REN* expression was associated with poor prognosis in THYM, KIRP, BRCA-LumA, and ACC. In JGCTs, *REN* serves as a central component of the renin-angiotensin system (RAS) and plays a role in blood pressure regulation. Emerging evidence also suggests that REN activation contributes to tumor initiation, progression, and modulation of the tumor immune microenvironment beyond its canonical role in blood pressure control. More importantly, except for PDGFB attributed to *REN* secreting, the PPARG pathway (4) and NOTCH signaling (22) have also been implicated in REN production. Pathway enrichment analysis confirmed activation of both pathways in juxtaglomerular cells. In summary, we characterized the immune microenvironment of JGCTs and identified high *KISS1* expression as a potential contributor to tumor development.

## Conclusion

This study presents the first case of JGCT in an 8-year-old girl in China, with successful recovery after partial nephrectomy. We also performed the first spatial transcriptomic analysis of reninoma, providing new insights into the molecular biology of human JGCTs. *KISS1* was identified as a novel candidate gene potentially involved in JGCT development. In addition, the PPARG, NOTCH, and PDGFB pathways emerged as potential regulators of renin secretion.

## Data Availability

The original contributions presented in the study are included in the article material. Further inquiries can be directed to the corresponding author. Publicly available datasets were analyzed in this study. Spatial sequencing data were available from figshare database. https://figshare.com/s/e9eea249aa64a17aee08.
